# Bioactive Flavonoids, Antioxidant Behaviour, and Cytoprotective Effects of Dried Grapefruit Peels (*Citrus paradisi* Macf.)

**DOI:** 10.1155/2016/8915729

**Published:** 2016-01-21

**Authors:** Lucia Castro-Vazquez, María Elena Alañón, Virginia Rodríguez-Robledo, María Soledad Pérez-Coello, Isidro Hermosín-Gutierrez, María Consuelo Díaz-Maroto, Joaquín Jordán, María Francisca Galindo, María del Mar Arroyo-Jiménez

**Affiliations:** ^1^Analytical Chemistry and Food Technology Area, Faculty of Pharmacy, University of Castilla-La Mancha, 02071 Albacete, Spain; ^2^Food Technology Area, Faculty of Chemistry, University of Castilla-La Mancha, 02071 Ciudad Real, Spain; ^3^Medical Sciences Department, Faculty of Medicine, University of Castilla-La Mancha, 02071 Albacete, Spain

## Abstract

Grapefruit (*Citrus paradisi* Macf.) is an important cultivar of the* Citrus* genus which contains a number of nutrients beneficial to human health. The objective of the present study was to evaluate changes in bioactive flavonoids, antioxidant behaviour, and* in vitro* cytoprotective effect of processed white and pink peels after oven-drying (45°C–60°C) and freeze-drying treatments. Comparison with fresh grapefruit peels was also assessed. Significant increases in DPPH, FRAPS, and ABTS values were observed in dried grapefruit peel samples in comparison with fresh peels, indicating the suitability of the treatments for use as tools to greatly enhance the antioxidant potential of these natural byproducts. A total of thirteen flavonoids were quantified in grapefruit peel extracts by HPLC-MS/MS. It was found that naringin, followed by isonaringin, was the main flavonoid occurring in fresh, oven-dried, and freeze-dried grapefruit peels.* In vivo* assay revealed that fresh and oven-dried grapefruit peel extracts (45°C) exerted a strong cytoprotective effect on SH-SY5Y neuroblastoma cell lines at concentrations ranging within 0.1–0.25 mg/mL. Our data suggest that grapefruit (*Citrus paradisi* Macf.) peel has considerable potential as a source of natural bioactive flavonoids with outstanding antioxidant activity which can be used as agents in several therapeutic strategies.

## 1. Introduction

Today, there is increasing demand for natural bioactive compounds as people express more concern about their health, especially in connection with health-giving diets. Epidemiological studies suggest that high dietary intake of phytochemicals, in particular of polyphenols, is associated with a reduced risk of a multitude of chronic diseases.

In this connection, fruits of the* Citrus* genus are recognized as being a healthful source of bioactive compounds such as vitamins, carotenoids, fibre, and phenolic compounds [1–3]. Worldwide agricultural citrus production, including oranges, mandarins, lemons, bergamots, limes, pummelos, and grapefruits, has been increasing strongly in the last decades, reaching over 100 million metric tons per year [4]. About a third of citrus fruits go to produce fresh juice or citrus-based drinks. The juice yield of citrus fruits accounts for half of the fruit weight, and hence a very large amount of pulp and peel waste is produced worldwide every year [5].

It has been found that peels are the main sources of polyphenols in citrus fruits [6]. Peel residues from sweet and bitter oranges, lemons, and mandarins have proved to be an important source of phenolic acids and flavonoids, chiefly polymethoxyflavones (PMFs), flavanones, and glycosylated flavanones [7–10]. These bioactive compounds are strongly associated with therapeutic properties including antiallergenic, antiatherogenic, anti-inflammatory, antimicrobial, anticarcinogenic, antithrombotic, cardioprotective, and vasodilatory effects [11–18]. Many of these pharmacological activities of citrus polyphenols are a consequence of their ability to scavenge reactive oxygen species (ROS) and reactive nitrogen species (RNS) [19].

Since oxidative stress is involved in all the above-mentioned pathological conditions, the outstanding antioxidant role of natural polyphenols has received much attention from many researchers. In this regard,* Citrus* flavonoids have recently attracted considerable interest as potential therapeutic agents in numerous* in vitro* and* in vivo* studies. Naringin, high levels of which occur in several varieties of citrus fruits and citrus byproducts, has demonstrated anti-inflammatory, anticarcinogenic, lipid-lowering, and antioxidant activities [20–23]. Hesperidin, one of the main flavanone glycosides, which occurs in oranges, has been shown to exert a wide range of therapeutic effects such as antioxidant, anti-inflammatory, and anticarcinogenic properties [20]. Moreover, it has been found to significantly reduce ROS generation in cells [23, 24] and to restore mitochondrial enzyme activity [25].

Citrus flavonoids may also exert neuroprotective effects since they are involved in the modulation of neuronal activities and mental health including brain plasticity, behaviour, mood, depression, and cognition [20, 22]. In this regard, it has been demonstrated that hesperidin can protect neurons against various types of insults associated with many neurodegenerative diseases [26]. Also, naringin has proven to exert neuroprotective effects through anti-inflammatory activity on the survival of dopaminergic neurons and on the integrity of the nigrostriatal pathway in animal models of Parkinson's disease [27–29]. Natural flavonoids would therefore seem to have important potential as medicaments in the field of mental health, although their use in clinical practice is still a long way off [30].

The peel from* Citrus* fruits is also a source of Polymethoxylated Flavones (PMFs), flavonoids substituted by methoxy groups, which rarely occur in other plants [31]. PMFs are more physiologically active than their methylated derivatives. For instance, research data have demonstrated that nobiletin possesses a wide range of therapeutic applications including antioxidant, antitumor properties, in both* in vitro* and* in vivo* models [32–36]. Moreover, it has recently been reported that a novel citrus tangeretin derivative, 5-acetyl-6,7,8,4′-tetramethylnortangeretin, can inhibit MCF-7 breast cancer cell proliferation [37]. These data provide new insights into the role that citrus polyphenols can play in the prevention of diseases.

In recent years, white and pink grapefruits (*Citrus paradisi* Macf.) have attracted much attention because of their nutritional and antioxidant properties [38]. High levels of bioactive flavanones glycosides, namely, naringin and narirutin, have been reported in seed and peel residues released after grapefruit juice extraction [38, 39], although further research is required to explore the composition of this fruit variety and its byproducts in more detail.

Several treatments, including far-infrared radiation, ultrasound-assisted alkaline hydrolysis, enzyme treatment, and heat treatment, have been proposed to release more bioactive glycosylated flavonoids and low molecular weight phenolic compounds from several species of citrus genus [1, 4, 40]. In this connection it has been found that dried orange, mandarin, and lemon peel extracts contained much higher concentrations of phenolic compounds than fresh ones and hence exhibited greater antioxidant activity [14, 41, 42].

However, little is known about the bioactive flavonoids in treated grapefruit (*Citrus paradisi* Macf.) peel as a source of health-promoting phytochemicals. To our knowledge, only Xu et al. 2007 [42] have discussed the extractable phenolic fraction of grapefruit (*Citrus paradisi Changshanhuyou*), in a cultivar located in southern China, showing increases in the individual phenolic compounds and enhancements of antioxidant capacity after heat treatment.

Based on these results, then, it is easy to understand the interest of new comprehensive studies to determine the potential of treated grapefruit (*Citrus paradisi* Macf.) peel as a natural product that can serve as an outstanding low-cost antioxidant source. Treated grapefruit peel residues could play an important role in the development of nutraceutical products or as therapeutic agents for use in various pharmacological* in vitro* or* in vitro* approaches.

For all these reasons, the objectives of this research were as follows: (i) to describe and quantify the flavonoid profiles and antioxidant activities of processed white and pink grapefruit peels (*Citrus paradise* Macf.) after oven-drying and freeze-drying; (ii) to study the* in vitro* cytoprotective effectiveness of grapefruit peel extracts on SH-SY5Y neuroblastoma cell lines.

## 2. Material and Methods

### 2.1. Fruit Peel Materials

White and pink grapefruits (*Citrus paradise*) were grown in several Valencia areas (Spain) corresponding with the cropping areas. They were purchased at Corte Ingles supermarket and possess certified geographical origin.

### 2.2. Oven-Dried and Freeze-Dried Grapefruit Peels Treatment

Grapefruits were cleaned with distilled water in the laboratory and they were immediately peeled. White and pink grapefruits peels were cut into pieces (sized from approximately 0.5 × 0.5 cm thick). Sliced peel was divided into four portions: (i) one fresh peel portion to be directly analyzed; (ii) two fractions which were dried in an oven at 45°C and 60°C, respectively, until their water content was within 9–12%; (iii) one grapefruit peel fraction that was freeze-dried.

### 2.3. Extraction of Phenolic Compounds

Extraction of grapefruits peels was carried out by means of an accelerated solvent extractor ASE 200 (Dionex Corp, Sunnyvale, CA, USA). Extractions were performed using 5 g of grapefruits peel which was placed into inox extraction cells of 22 mL. Every cell was filled with methanol and raised to 60°C. Then, two static extraction phases lasting for 10 min were carried out under 1500 psi. Between extractions, a rinse of the complete system was performed to avoid any carry-over.

Extracts were evaporated using a rotavapor with a vacuum controller (Heidolph, Schwabach, Germany) at 40°C. Samples were redissolved with 5 mL of methanol and they were filtered through a Whatman Number 1 filter paper. Samples were kept at −20°C prior to being used to determine antioxidant activity and phenolic compounds.

### 2.4. HPLC-DAD-ESI-MS Analysis

HPLC separation and identification and quantification of phenolic compounds were performed on an Agilent 1100 series system (Agilent, Waldbronn, Germany), equipped with a DAD photodiode detector (G1315B) and a LC/MSD Trap VL (G2445C VL) electrospray ionization mass spectrometry (ESI/MSn) system, both coupled to an Agilent Chem Station (version B.01.03) for data processing.

The samples, after filtration (0.20 *μ*m, polyester membrane, Chromafil PET 20/25, Macherey-Nagel, Düren, Germany), were injected in duplicate on a reversed-phase narrow-bore column Zorbax Eclipse XDB-C18 (2.1 × 150 mm; 3.5 *μ*m particle; Agilent) protected by a guard column Zorbax Eclipse XDB-C8 (2.1 × 12.5 mm; 5 *μ*m particle; Agilent), both thermostated at 40°C.

The solvents were as follows: solvent A (acetonitrile/water/formic acid, 3 : 88.5 : 8.5, v/v/v), solvent B (acetonitrile/water/formic acid, 50 : 41.5 : 8.5, v/v/v), and solvent C (methanol/water/formic acid, 90 : 1.5 : 8.5, v/v/v). The flow rate was 0.190 mL/min. The linear solvents gradient was as follows: 0 min, 99% A and 1% B; 8 min, 97% A and 3% B; 37 min, 70% A, 17% B, and 13% C; 40 min, 50% A, 30% B, and 20% C; 51 min, 10% A, 40% B, and 50% C; 56 min, 50% B and 50% C; 59 min, 50% B and 50% C; and 65 min, 99% A and 1% B.

For identification, ESI-MSn was used in both positive and negative modes, setting the following parameters: dry gas, N2, 11 mL/min; drying temperature, 350°C; nebulizer, 65 psi; capillary, −2500 V (positive ionization mode) up to 42 minutes and +2500 V (negative ionization mode) until the end of the chromatogram; target mass, 600 *m*/*z*; compound stability, 40% (negative ionization mode) and 100% (positive ionization mode); trap drive level, 100%; and scan range, 50–1200 *m*/*z*.

The identification of flavonoid compounds was carried out by comparing their retention times and mass spectra provided with those of authentic standard (from Sigma St. Louis, MO) when available and spilling the samples with standard solutions. This was the case of hesperidin, neohesperidin, naringin, naringenin, nobiletin, and tangeretin.

The identification of compounds where the standards were not available was performed by comparing the UV spectra and the [M + H]^+^, [M − H]^−^ *m*/*z* with those reported in the literature. Quantification was made by means of external standard calibration lines and was expressed as milligrams of compounds per gram of dry weight (DW). Quantitative results for compounds without chemical standard were expressed in mg·g^−1^ naringin equivalents.

### 2.5. DPPH Radical Scavenging Assay

The DPPH assay was carried out according to the method proposed by [43] where 1,1-diphenyl-2-picrylhydrazyl radical was used as a stable radical. One hundred microliters of different dilutions of extracts was added to 2.9 mL of a 0.06 mM methanol DPPH radical solution. Methanol was used to adjust the zero and the decrease in absorbance was measured at 515 nm every minute for 25 min in a UV-vis spectrophotometer (Helios, Thermo Spectronic, Cambridge, UK). Only values between 20% and 80% of the initial absorbance of the radical DPPH were taken into consideration. Concentrations were calculated from a calibration curve in the range between 0.1 and 0.8 mM trolox. Results were expressed in milligrams of trolox per gram of dry weight.

### 2.6. ABTS^∙+^  Radical Scavenging Assay

The method used was the ABTS^∙+^  (radical cation azino-bis[3-ethylbenzthiazoline-6-sulfonic acid]) decolourisation assay according to [44]. The assay is based on the ability of an antioxidant compound to quench the ABTS^∙+^  relative to that of a reference antioxidant such as trolox. A stock solution of ABTS^∙+^  radical cation was prepared by mixing ABTS solution and potassium persulfate solution at 7 mM and 2.45 mM final concentration, respectively. The mixture was maintained in the dark at room temperature for 12–16 h before use. The working ABTS^∙+^  solution was produced by dilution in ethanol (1 : 90 v/v) of the stock solution to achieve an absorbance value of 0.7 (±0.02) at 734 nm. An aliquot of 20 *μ*L of diluted extract was added to ABTS^∙+^  working solution (3 mL). For the blank and standard curve, 20 *μ*L of ethanol or trolox solution was used, respectively. Absorbance was measured by means of a UV-vis spectrophotometer at 734 nm immediately after addition and rapid mixing (*At* = 0) and then every minute for 5 min. Readings at *t* = 0 min (*At* = 0) and *t* = 5 min (*At* = 5) of reaction were used to calculate the percentage inhibition value for each extract.

A standard reference curve was constructed by plotting % inhibition value against trolox concentration (0.1–0.8 mM). The radical scavenging capacity of extracts was quantified as milligrams of trolox per gram of dry weight.

### 2.7. FRAP Assay

The FRAP assay (Ferric Reducing Ability of Plasma) was performed as previously described by Alañón et al. (2011a) and Benzie and Strain, 1999 [43, 45], with some modifications. This spectrophotometric assay measures the ferric reducing ability of antioxidants. The experiment was conducted at 37°C and pH 3.6. In the FRAP assay, antioxidants present in the extract reduce Fe (III)-tripyridyltriazine complex to the blue ferrous form, with an absorption maximum at 593 nm. The assay was performed by means of an automated microplate reader (Tecan GENios Pro (Tecan Ltd., Dorset, UK)) with 96-well plates. Reagents included 300 mM acetate buffer pH 3.6, 40 mM hydrochloric acid, 10 mM TPTZ solution, and 20 mM ferric chloride solution. The working FRAP reagent was prepared fresh on the day of analysis by mixing acetate buffer, TPTZ solution, and ferric chloride solutions in the ratio 10 : 1 : 1 and the mixture was incubated at 37°C. Diluted extract (30 *μ*L) and prewarmed FRAP reagent (225 *μ*L) were put into each well. The absorbance at time zero and after 4 min was recorded at 593 nm. The calculated difference in absorbance is proportional to the ferric reducing/antioxidant power of the extract. For quantification, a calibration curve of trolox was prepared with dilutions within 0–1.5 mM. The final results were expressed as milligrams of trolox per gram of dried grapefruit peel.

### 2.8. Total Phenol Index (TPI)

The total phenol content of extracts was determined according to the Folin-Ciocalteu procedure described by Singleton and Rossi [46]. Deionized water (1.8 mL) was added to 0.2 mL of each extract. Folin-Ciocalteu reagent (0.2 mL) was then added and tubes were shaken vigorously. After 3 min, 0.4 mL sodium carbonate solution (35% w/v) was added, along with 1.4 mL of deionized water. Samples were well mixed and left in the dark for 1 h. The absorbance was measured at 725 nm using a UV-vis spectrophotometer (Lambda 5, Perkin-Elmer, Seer Green, UK) and the results were expressed in gallic acid equivalents, GAE, using a gallic acid standard curve (0–0.2 mg mL^−1^). Extracts were further diluted if the absorbance value measured was above the linear range of the standard curve.

### 2.9. Cell Culture and Drug Treatment Procedures

SH-SY5Y cultures were grown as described previously by Jordán et al., 2004 [47], in Dulbecco's modified Eagle's medium (DMEM) supplemented with 2 mM L-glutamine, 20 units·mL^−1^ penicillin, 5 mg·mL^−1^ streptomycin, and 15% (v/v) fetal bovine serum (Invitrogen, Carlsbad, CA, USA). The SH-SY5Y cells (1 × 10^6^/mL) were seeded 24 h before the experiments in a 96-well plate and they were grown in a humidified cell incubator at 37°C under a 5% CO_2_ atmosphere. For treatments, extracts from white and pink grapefruit peels (fresh, dried, and freeze-dried) were directly added to the culture medium at different concentrations (0.1, 0.25, 0.50, 0.75, and 1 mg/mL) for 24 h. The corresponding controls were treated with the same concentration of ethanol, which was always below 0.1% (final concentration).

### 2.10. Viability Assay: 3-(4,5-Dimethylthiazol-2-yl)-2,5-diphenyltetrazolium Bromide (MTT) Assay

Cell viability was measured by the ability to reduce 3-(-4,5-dimethylthiazol-2-yl)-2,5-diphenyltetrazolium bromide (MTT) to the blue formazan product. The culture medium was removed after 24 h of treatment. 150 *μ*L of MTT (1 mg·mL^−1^ in normal culture medium) was added to the plates, and the cells (control and treated) were incubated for 2 h at 37°C. The medium was then replaced with DMSO, and MTT absorption was measured in a VERSAmax tunable microplate reader (Molecular Devices, Sunnyvale, CA, USA). Results were expressed as the percentage of MTT reduction, assuming that the absorbance of the control SH-SY5Y cells was 100%.

### 2.11. Statistical Analysis

Analysis of variance and multivariate analysis were performed using* SPSS* 15.0 for Windows statistical package. Differences among means were determined for significance at *p* ≤ 0.05 using the Student-Newman-Keuls test. Principal Component Analysis was performed to classify the samples into groups according to phenolic composition and antioxidant activity.

## 3. Results and Discussion

### 3.1. Effect of Processing on the Antioxidant Activities and Total Polyphenol Index of Grapefruit Peel Extracts

The effects of oven-drying and freeze-drying treatments on the antioxidant activity of grapefruit peels extracts were determined by DPPH, ABTS^∙+^, and FRAPS tests (see Table 1). It is interesting to note that freeze-drying enhanced antioxidant activity in all cases.

The DPPH assay showed significantly higher levels of antioxidant capacity (*p* < 0.05) in freeze-dried than in fresh grapefruit peels. Freeze-dried white and pink grapefruit peels registered 122.83 and 110.98 mg trolox/g DW, while extracts from fresh peels registered values of 32.46 and 25.17 mg trolox/g DW, respectively. This effect is probably a consequence of the freeze-drying process. This process has been associated with high production of redox-active metabolites which play an important role in adsorbing and neutralizing free radicals or decomposing peroxides, as previously reported by other researchers [48].

After oven-drying at 45°C and 60°C, DPPH values of both white and pink grapefruit peels were significantly greater than those of fresh peel extracts (Table 1). These increases denote increased antioxidant activity, particularly in the case of white grapefruit peel heated at 60°C. They are presumably a consequence of the relationship between the generation of breakdown antioxidant products and the increasing temperatures to which the grapefruit peels were subjected, and they are consistent with data reported for other citrus varieties subjected to comparable heat treatments [41, 42, 49]. The ABTS^∙+^  assay showed the same tendency, revealing a significant increase of free radical scavenging activity in white and pink freeze-dried grapefruit peel extracts (*p* < 0.05), which reached 537.45 and 455.38 mg trolox/g DW, versus 122.34 and 99.46 mg trolox/g DW in the case of fresh samples.

ABTS^∙+^  activity was also greater in grapefruit peel dried at 45°C and 60°C than in fresh extracts, although the rise was less pronounced than in the case of freeze-dried peel. For instance, scavenging ability was significantly (*p* < 0.05) greater in white grapefruit peel extracts than in fresh extracts (from 122.34 to 194.81 and 339.66 mg trolox/g DW) as a result of treatment at 45°C and 60°C, respectively. ABTS also increased in the case of pink grapefruit peel but did not differ significantly between 45°C and 60°C. This behaviour is consistent with reports for extracts of dried citrus peel of other varieties [42, 49].

FRAP chelating values for white and pink grapefruit peels oven-dried at 60°C reached 105.86 and 79.43 mg trolox/g DW, respectively, that is, 1.7 times the values for fresh peels extracts (Table 1). In the case of freeze-dried samples, results varied from 60.30 and 44.82 for white and pink fresh grapefruit peel extracts to 181.80 and 207.74 mg trolox/g DW for freeze-dried grapefruit peels, respectively.

In general, the scale of the antioxidant activity observed in freeze-dried white and pink grapefruit peels suggested that this treatment might produce not only dissociation or liberation of some phenolic compounds from biological structures but also chemical changes enabling the conversion of insoluble phenols into more soluble and free forms, as indicated by the data from other freeze-dried vegetable extracts [50, 51].

The results of the Folin-Ciocalteu Total Phenol Index (TPI), a preliminary screening factor to establish the antioxidant capacities of treated grapefruit peels, are shown in Table 1. Total phenols in white and pink grapefruit peels dried at 45°C, and particularly at 60°C (63.35 and 49.36 mg GAE/g dry weight, resp.), were significantly higher than in fresh samples (49.14 and 27.95 mg GAE/g dry weight), most probably due to the cleaving of glycosylated bonds in various phenolic compounds.

Note also that TPI values from white and pink freeze-dried grapefruit peel extracts were 58% and 42% higher, respectively, than in fresh samples. This trend is consistent with reports in previous works carried out on lyophilized fruits, tubers, vegetables, and fungi [50, 52, 53].

The same trend was observed in the behaviour of TPI, ABTS, FRAP, and DPPH values, which were significantly enhanced after oven-drying (45°C, 60°C) and especially freeze-drying treatments. This suggests that both processes could be successfully used to enhance antioxidant activity in grapefruits peels for use as natural sources of antioxidants, with major attendant environmental and economic benefits. As natural products, with outstanding antioxidant power, processed grapefruit look very promising for use in the development of new therapeutic strategies.

### 3.2. Effect of Oven-Drying and Freeze-Drying on Flavanone and Polymethoxylated Flavones of Grapefruit Peels

**Figure 1:**
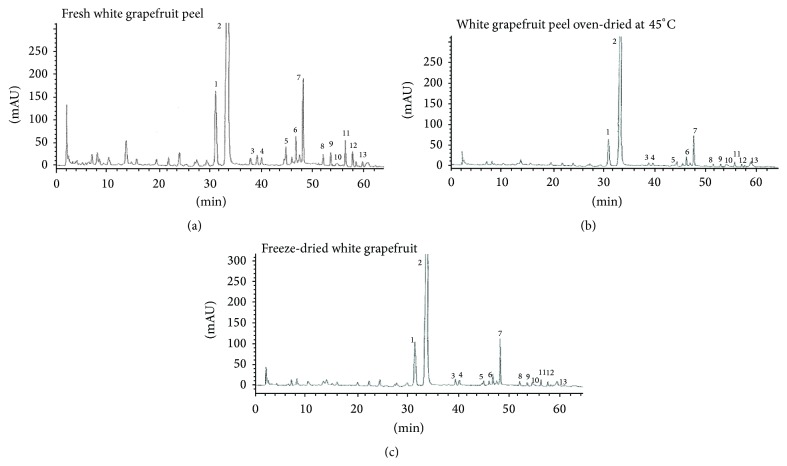
HPLC chromatogram of white grapefruit peels. Peak assignments: (1) isonaringin; (2) naringin; (3) hesperidin; (4) neohesperidin; (5) unknown-1; (6) unknown-2; (7) naringenin; (8) hesperetin; (9) isosinensetin; (10) sinensetin; (11) unknown-3; (12) nobiletin; (13) tangeretin.

A total of thirteen compounds were quantified, based on their UV-data spectra and [M + H]^+^, [M − H]^−^ *m*/*z* (Table 2), and were quantified by HPLC-MS. These were as follows: four glycosylated flavanones (FGs), namely, isonaringin, naringin, hesperidin, and neohesperidin; four polymethoxylated flavones (PMFs), namely, isosinensetin, sinensetin, nobiletin, and tangeretin; two flavanone aglycones: hesperetin and naringenin; and three unknown compounds. Typical MS total ion current chromatograms with numbered peaks are shown in Figure 1.

Naringin and to a much lesser extent isonaringin were the main FGs in all grapefruit peels extracts (Table 3). Naringin levels in fresh pink and white grapefruits varied within 142–160 mg/g DW, respectively, while isonaringin ranged between 11.85 and 13.42 mg/g DW. Moreover, naringenin was the most abundant flavanone aglycone in fresh white and pink grapefruit peels (Table 3).

Levels of polymethoxylated flavones, sinensetin, nobiletin, and tangeretin ranged from 1.03 to 3.45 mg/g DW in fresh grapefruit peels. Results were very similar to reports in the literature for peels of mandarin and thirteen citrus species [53–56]. However, one of the most outstanding findings in this study was the flavonoid losses in processed grapefruit peels. Oven-dried and freeze-dried grapefruit peels had similar flavonoid profiles, although concentrations were lower than in fresh samples. After oven-drying of grapefruit peels at 45°C and 60°C, the concentrations of most FGs, PMFs, and flavanone aglycones declined sharply, 3–6-fold with respect to fresh grapefruit peel extracts (Table 3). The same behaviour has been reported in dried citrus peels from other varieties [42, 49]. However, it is important to note that naringin concentrations in processed grapefruit peels were between two and four times higher than reported in treated byproducts from other citrus species [42, 57, 58].

The same downward trend was observed when comparing flavonoid contents in freeze-dried grapefruit peels and fresh grapefruit peel extracts (Table 2). For instance, isonaringin decreased from 13.42 mg/g DW in fresh white grapefruit peels to 7.17 mg/g DW in freeze-dried white grapefruit peels. The decreases were most pronounced (from 13.42 to 4.57 and 4.02) after oven-drying at 45°C and 60°C, respectively.

It is interesting to note that the effects of oven-drying and freeze-drying on FG and PMF levels were opposite to their effects on TPI values and free radical scavenging activities. This may be because the TPI assay evaluates the totality of phenols, that is, all flavonoids and nonflavonoid phenolic compounds, which suggests that some phenolic compounds other than flavones and flavanones are involved in the antioxidant activities of grapefruit peel extracts. This tends to confirm some recent studies which described progressive increases of cinnamics and benzoics acids levels in orange peels dried from 60°C to 120°C in comparison with untreated samples due to the cleaving of esterified bond and glycosylated bond [42, 49]. By the other hand, our results are in a good agreement with decreases of naringin, hesperidin, and neohesperidin reported in dried orange peels in comparison with untreated samples [42]. Heat treatment of grapefruit peels is thus closely associated with releases of bound phenolic acids, including hydroxybenzoic and hydroxycinnamic acids, producing higher levels of free phenolic acids. Therefore, the increase in the total phenol index and DDP, FRAP, and ABTS values would appear to be caused by the free fraction of phenolic acids.

**Figure 2:**
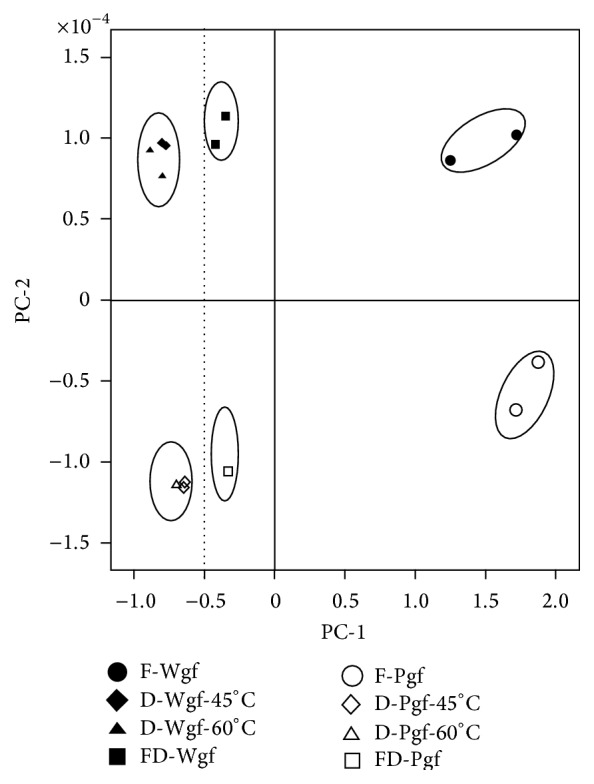
Principal Component Analysis performed considering duplicates of the flavanones glycosides, flavones, and polymethoxylated flavones contents on fresh, oven-dried and freeze-dried, and white and pink grapefruit peel extracts. F-Wgp: fresh white grapefruit; F-Pgf: fresh pink grapefruit; D-Wgf-45°C: dried white grapefruit at 45°C; D-Pgf-45°C: dried pink grapefruit at 45°C; D-Wgf-60°C: dried white grapefruit at 60°C; D-Pgf-60°C: dried white grapefruit at 60°C; FD-Wgf: freeze-dried white grapefruit; FD-Pgf: freeze-dried pink grapefruit.

To obtain more detailed information on the individual flavanone glycosides, flavanones, and polymethoxylated flavones that occur in processed white and pink grapefruit peels, the data matrix was processed by Principal Component Analysis (PCA). The two-dimensional projection of variables is presented in Figure 2.

The first principal component axis explains 83.47% of the total variation and clearly isolated fresh grapefruit peel extracts which were grouped on the right side of the plot, correlating closely with higher levels of hesperidin, neohesperidin, isonaringin, naringin, nobiletin, and unknowns-1-2.

PC-1 also exhibited correlation with freeze-dried samples plotted on the negative area of PC-1 (Figure 2). The fact that amounts of isosinensetin, hesperetin, hesperidin, isonaringin, neohesperidin, unknown-3, naringin, and nobiletin in freeze-dried grapefruit peels were higher than in extracts from oven-dried peels indicates a good degree of discrimination and also suggests that freeze-drying is more effective in preserving bioactive compounds than oven-drying. However, extracts from grapefruit peels dried at 45°C and 60°C were located too close together on the *x*-axis for discrimination.

PC-2 explains 10.04% of the total variation and is particularly important in terms of differentiating grapefruit varieties. PC-2 showed positive loadings for tangeretin, sinensetin, and naringenin, grouping white grapefruit peel extracts at the top of the axis and pink grapefruit peel extracts at the bottom.

### 3.3. Viability

SH-SY5Y cell viability results were influenced by two parameters; firstly the treatment of grapefruit peels (fresh, oven-drying, or freeze-drying) and secondly the concentration of bioactive compounds. Our results showed that the potential cell protection and/or cell cytotoxicity of grapefruit peel extracts was determined by these two factors together.

In general, cell viability decreased with increasing concentrations of grapefruit peel extracts, whether they were fresh/processed or white/pink. On the other hand, in the case of SH-SY5Y cells viability percentages were higher in fresh than in treated grapefruit peel extracts.

**Figure 3:**
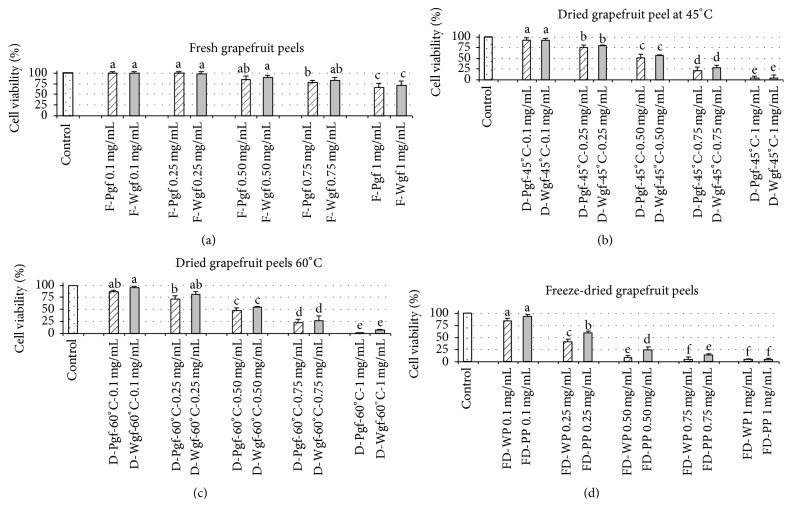
Effects of different concentrations of red and white grapefruit extracts in cell viability of SH-SY5Y cell cultures for 24 h. Data were expressed as the percentage of live cells relative to total cells. Data are presented by means ± SD (*n* = 3). F-Wgp: fresh white grapefruit; F-Pgf: fresh pink grapefruit; D-Wgf-45°C: dried white grapefruit at 45°C; D-Pgf-45°C: dried pink grapefruit at 45°C; D-Wgf-60°C: dried white grapefruit at 60°C; D-Pgf-60°C: dried white grapefruit at 60°C; FD-Wgf: freeze-dried white grapefruit; FD-Pgf: freeze-dried pink grapefruit. a, b, c, d, e, f: different letters in the same column denote a significant difference according to the Student-Newman-Keuls test, at *p* < 0.05.

It is important to stress that fresh white grapefruit peel extracts at concentrations between 0.1 and 0.25 mg/mL clearly exerted a protective effect on the SH-SY5Y cell line, reaching viabilities of 100% (Figure 3). Similar effects were reported by Chen et al. 2012 [59] on Hep G2 cells after contact with fresh* Citrus sinensis* peel extracts at concentrations ranging within 0.01–0.5 mg/mL, which significantly protected against tertiary butyl hydroperoxides t-BHP.

The cytoprotective effect observed in the current is most probably attributable to the levels of bioactive flavonoids (FGs and PMFs), mainly naringin, isonaringin, and naringenin, which largely occur in fresh white grapefruit peel extracts (Table 2). Similar finding have also been reported, revealing a relationship between naringin and naringenin and neuroprotection and oxidative stress delay [60]. In the present case, fresh white grapefruit peel extracts, which registered the highest flavonoid contents, also scored best for viability. On the other hand, freeze-dried peel extracts, containing less flavonoids (Table 2), registered the lowest cell viability ratios.

Also, SH-SY5Y cell viability decreased to 75% after the following: (i) 24-hour incubation with fresh grapefruit peel extracts at 0.75 mg/mL; (ii) incubation with oven-dried grapefruit peels extract (45°C and 60°C) at 0.25 mg/mL; and (iii) contact with freeze-dried grapefruit peel extract at concentrations ranging within 0.1–0.25 mg/mL (Figure 3).

Finally, it was found that oven-dried grapefruit peels (45°C and 60°C), at concentrations ranging from 0.75 mg/mL to 1 mg/mL, induced cell death by 75–95% in both white and pink grapefruits. This effect was especially pronounced after cell contact with freeze-drying extracts (0.75 mg/mL and 1 mg/mL) which triggered 90% and 96%, respectively, of apoptosis in SH-SY5Y-cells. This suggests that cell cytoprotection and/or apoptosis, expressed as cell viability, can be influenced in a dose-dependent way by flavonoids.

It should be noted that the results for SH-SY5Y cell viability after contact with grapefruit peel extracts did not really match expectations in the light of the polyphenol index (TPI) and DPPH, FRAP, and ABTS here reported. There seemed to be an inverse correlation between antioxidant activity and cell viability since the extracts with the highest antioxidant capacities were the most closely associated with cell cytotoxicity. The explanation of this singular behaviour probably lies in the amounts of phenolic acids, which would surely increase after drying at 45°C and 60°C and more so after freeze-drying, producing considerable increases in the overall antioxidant activity and TPI index. However, it has also been reported that phenolic acids, due to their chemical structure and depending on certain conditions, are involved in prooxidant reactions associated with damage to molecules such as DNA [61, 62]. These last findings would seem to indicate that phenolic acid levels are strongly associated with cell cytotoxicity and apoptosis, which supports the findings in the present work.

## 4. Conclusions

Our results indicate that oven-drying (45°C, 60°C) and especially freeze-drying can be used to significantly enhance the antioxidant power of grapefruit peels, thus realizing their outstanding potential for biomedical use.

Fresh and processed grapefruit peel wastes are a natural source of valuable bioactive flavonoids, mostly naringin, that could be incorporated as food ingredients or as therapeutic agents as part of pharmacological strategies.

Finally, the* in vitro* cytoprotection demonstrated by fresh and oven-dried (45°C) grapefruit peels opens up new possibilities for these natural extracts; however, further research into action mechanisms, animal models, clinical trials, and dose-effect will be needed.

## Figures and Tables

**Table 1:** Total Polyphenol Index (TPI), DPPH, FRAP, and ABTS assays mean values and standard deviation (SD) of untreated, oven-dried, and freeze-dried grapefruit peel extracts.

	TPI	DPPH	FRAP	ABTS
	Mean ± SD	Mean ± SD	Mean ± SD	Mean ± SD
Fresh white grapefruit	49.14^a^ ± 7.91	32.46^a^ ± 0.80	60.30^a^ ± 3.25	122.34^a^ ± 6.22
Fresh pink grapefruit	27.95^b^ ± 0.83	25.18^a^ ± 8.52	44.82^b^ ± 5.35	99.46^a^ ± 12.09
White grapefruit dried at 45°C	52.95^a^ ± 4.83	48.05^b^ ± 3.75	71.57^a^ ± 0.60	194.81^b^ ± 3.80
Pink grapefruit dried at 45°C	42.29^a^ ± 3.30	35.26^a^ ± 3.62	65.86^a^ ± 5.28	175.87^b^ ± 5.64
White grapefruit dried at 60°C	63.35^c^ ± 0.84	86.76^c^ ± 8.40	105.86^c^ ± 22.39	339.66^c^ ± 33.61
Pink grapefruit dried at 60°C	49.36^ac^ ± 3.57	50.07^b^ ± 2.26	79.43^ac^ ± 5.16	210.78^b^ ± 2.19
Freeze-dried white grapefruit	84.60^d^ ± 10.80	122.83^d^ ± 15.95	181.80^d^ ± 25.97	537.48^d^ ± 36.10
Freeze-dried pink grapefruit	66.70^e^ ± 1.54	110.98^d^ ± 13.76	207.74^d^ ± 14.65	455.38^e^ ± 1.95

TPI are expressed as mg gallic acid equivalents per gram of dry weight.

DPPH, FRAP, and ABTS assays are expressed as mg trolox per gram of dry weight.

^a,b,c,d,e^Different letters in the same column denote a significant difference according to the Student-Newman-Keuls test, at *p* < 0.05.

**Table 2:** Spectral data of flavonoids in grapefruit peel extracts.

Tentative identification	RT	UVmax	MS [M − H]^−^	MS [M + H]^+^	Products ions
	(min)	*λ* (nm)	(*m*/*z*)	(*m*/*z*)	(*m*/*z*)
Isonaringin [32, 41]	31.43	217, 284, 331	579		271, 151
Naringin [32, 41]	33.58	224, 283, 331	579		459, 271
Hesperidin [32, 41]	38.02	225, 284, 328	609		301
Neohesperidin [41]	40.19	228, 283, 331	609		301, 489
Unknown-1	44.65	283, 328		617	465, 303
Unknown-2	46.57	249, 257, 324		595	449, 287
Naringenin [42]	48.04	226, 284, 325		273	153
Hesperetin [40]	51.98	225, 285, 329		303	285
Isosinensetin [40]	53.46	249, 270, 342		373	357, 343, 327
Sinensetin [32, 40, 43]	54.54	243, 264, 333		373	358, 343, 312
Unknown-3	56.22	250, 272, 335		403	388, 373
Nobiletin [40, 43]	57.60	248, 268, 334		403	388, 373
Tangeretin [32, 40]	59.42	271, 322		373	358, 343, 325, 297

References given in brackets are taken from papers with matching spectral data:

Angeloni et al. 2012 [32]; Rivas et al. 2008 [40]; Jeong et al. 2004 [41]; Xu et al. 2007 [42]; Alañón et al. 2011 [43].

**Table 3:** Flavanones glycosides, flavanones, and polymethoxylated flavones content (milligrams per gram of dry weight) and relative standard deviations (RSD) both for white and pink grapefruit peel extracts.

	Fresh white grapefruit		White grapefruit oven-dried at 45°C		White grapefruit oven-dried at 60°C		Freeze-dried white grapefruit		Fresh pink grapefruit		Pink grapefruit oven-dried at 45°C		Pink grapefruit oven-dried at 60°C		Freeze-dried pink grapefruit	
Isonaringin^*∗*^	13.42^a^	(13.92)	4.57^b^	(1.71)	4.02^b^	(0.22)	7.17^c^	(2.06)	11.85^a^	(10.73)	3.72^b^	(5.65)	3.55^b^	(0.32)	5.22^b^	(4.74)
Naringin	160.25^a^	(18.24)	59.41^b^	(1.96)	51.16^b^	(0.25)	95.08^c^	(5.39)	142.39^a^	(16.81)	45.92^b^	(6.39)	43.50^b^	(0.34)	50.40^b^	(3.12)
Hesperidin	3.23^a^	(15.81)	0.51^cd^	(2.20)	0.37^cd^	(5.82)	0.65^cd^	(1.90)	2.68^b^	(6.31)	0.21^d^	(6.29)	0.13^d^	(0.26)	0.95^c^	(2.77)
Neohesperidin	3.30^a^	(9.93)	0.79^c^	(2.34)	0.67^cd^	(0.48)	1.39^e^	(5.45)	2.93^b^	(7.40)	0.30^d^	(6.41)	0.27^d^	(0.39)	0.79^c^	(2.09)
Unknown-1^*∗*^	4.39^a^	(5.26)	0.76^c^	(2.17)	0.76^c^	(0.08)	0.74^c^	(1.26)	3.88^b^	(4.83)	0.93^c^	(10.65)	0.84^c^	(0.12)	0.61^c^	(5.59)
Unknown-2^*∗*^	5.36^a^	(6.71)	1.67^bc^	(2.28)	1.46^b^	(1.80)	2.20^c^	(7.01)	5.05^a^	(8.82)	1.60^bc^	(4.70)	1.29^b^	(0.08)	1.09^b^	(1.40)
Naringenin	8.49^a^	(9.95)	5.05^b^	(2.16)	4.54^b^	(1.10)	7.83^a^	(4.78)	8.14^a^	(15.83)	2.87^c^	(5.29)	2.49^c^	(0.29)	2.35^c^	(0.35)
Hesperetin^*∗*^	2.93^a^	(2.24)	0.54^c^	(1.94)	0.43^cd^	(1.17)	0.87^e^	(1.25)	2.55^b^	(3.03)	0.42^cd^	(5.57)	0.39^d^	(1.40)	0.47^cd^	(6.99)
Isosinensetin^*∗*^	3.02^a^	(3.71)	0.56^b^	(3.44)	0.45^b^	(0.59)	0.60^a^	(4.96)	3.12^a^	(10.67)	0.41^b^	(1.34)	0.43^b^	(2.07)	0.24^b^	(6.16)
Sinensetin^*∗*^	2.10^a^	(14.48)	1.82^a^	(0.13)	1.78^a^	(0.68)	1.74^a^	(2.68)	2.07^a^	(5.30)	0.63^b^	(0.77)	0.63^b^	(0.69)	0.79^b^	(7.69)
Unknown-3^*∗*^	4.07^a^	(9.42)	1.14^b^	(4.56)	0.99^b^	(0.87)	1.46^b^	(3.69)	3.86^a^	(9.83)	0.32^c^	(5.50)	0.32^c^	(3.68)	0.41^c^	(8.13)
Nobiletin	2.36^a^	(14.26)	1.22^c^	(1.29)	1.35^c^	(13.02)	2.09^a^	(12.63)	3.45^b^	(11.67)	0.63^d^	(2.74)	0.58^d^	(2.61)	1.24^c^	(10.19)
Tangeretin	2.45^a^	(10.48)	2.05^c^	(1.11)	1.98^c^	(5.29)	1.96^c^	(2.51)	1.03^b^	(3.34)	0.97^b^	(0.38)	0.97^b^	(0.46)	1.06^b^	(8.32)

^*∗*^Compounds tentatively identified. Data are expressed as naringin equivalents (mg/g).

^a,b,c,d,e^Different letters in the same column denote a significant difference according to the Student-Newman-Keuls test, at *p* < 0.05.
